# Complete Genome Sequence of Potential Deoxynivalenol-Degrading Bacterium Youhaiella tibetensis Type Strain F4

**DOI:** 10.1128/MRA.00984-19

**Published:** 2019-10-03

**Authors:** Yang Wang, Songxue Wang

**Affiliations:** a Academy of State Administration of Grain, Beijing, People’s Republic of China; Portland State University

## Abstract

Here, we report the first complete genome sequence of the potential deoxynivalenol-degrading strain Youhaiella tibetensis F4^T^ (China General Microbiological Culture Collection Center [CGMCC] 1.12719^T^). To the best of our knowledge, this is also the first announcement of the complete genome sequence of a *Youhaiella* species.

## ANNOUNCEMENT

The genus *Youhaiella* was first found in 2015 and is affiliated with the family *Hyphomicrobiaceae* and is a close relative of the genera *Paradevosia* and *Devosia* (1). It currently contains only one known species, Youhaiella tibetensis, and F4 is the type strain of the species. Recently, we found that the type strain F4, isolated from subsurface sediment (1), also possesses the ability to degrade deoxynivalenol (our unpublished results), as previously reported for *Devosia* spp. and Paradevosia shaoguanensis strains (2–5). To date, there has been no genome sequence reported for this microorganism. In the present study, we determined the complete genome sequence of *Y. tibetensis* F4^T^ for the purpose of determining the deoxynivalenol (DON) degradation gene and gaining a greater understanding of this strain.

The strain F4^T^ was purchased from the China General Microbiological Culture Collection Center (CGMCC). Total genomic DNA from a freshly grown R2A broth culture (1) was extracted using the Wizard genomic DNA purification kit (Promega) following the manufacturer’s instructions. A SMRTbell library was prepared using the SMRTbell template prep kit v1.0 (Pacific Biosciences), and genome sequencing was performed on the Pacific Biosciences RS II sequencing platform at Microanalysis Technology Co. Ltd. (Hefei, China). The sequencing reads (45,203), with an *N*_50_ value of 11,198 bp, were *de novo* assembled using the Hierarchical Genome Assembly Process v3.0 (HGAP) for assembly and Quiver for genome polishing in SMRT Analysis v2.3.0 (6). Default parameters were used for all software unless otherwise noted. Finally, the genome sequence of *Y. tibetensis* F4^T^ was assembled into a single circular chromosomal contig of 4,430,734 bp (64.92-fold coverage) with a mean G+C content of 64.8%. Gene prediction was based on using Prodigal v2.6.3 (7), Barrnap v0.8 (https://github.com/tseemann/barrnap), and tRNAscan-SE v2.0 (8). By these analyses, 4,348 protein-coding DNA sequence (CDS) genes, 48 tRNAs, and 2 rRNA operons, comprising 5S, 16S, and 23S rRNA genes, were detected in the genome. According to the annotation results, two genes involved in DON transformation were identified in the genome of *Y. tibetensis* F4^T^, including genes encoding pyrroloquinoline quinone (PQQ)-dependent alcohol dehydrogenase and one encoding aldo/keto reductase, which showed amino acid sequence similarities of 93.3% and 88.0% to the enzyme DepA (GenBank accession number WP_081840713) and DepB (GenBank accession number WP_035089809), respectively, in *Devosoia* sp. strain 17-2-E-8 (9, 10). The complete genome sequence of *Y. tibetensis* F4^T^ will help in elucidating the mechanisms of deoxynivalenol degradation in *Y. tibetensis*.

### Data availability.

The full genomic sequence of Youhaiella tibetensis F4^T^ has been deposited in NCBI/GenBank under BioProject number PRJNA553275, BioSample number SAMN12233448, SRA number SRR10083736, and accession number CP041690.
